# Molecular insights into the heterogeneity of depression in Alzheimer’s disease: A single‐cell transcriptomic analysis

**DOI:** 10.1002/alz.086507

**Published:** 2025-01-03

**Authors:** Michael W Lutz, Zhaohui Man, Ornit Chiba‐Falek

**Affiliations:** ^1^ Duke University School of Medicine, Durham, NC USA

## Abstract

**Background:**

Patients with Alzheimer’s Disease (AD) frequently manifest comorbid neuropsychiatric symptoms (NPS) with depression and anxiety being most prevalent. Previously we identified shared genetic risk loci between AD and major depressive disorder (MDD). In another study, we constructed a polygenic risk score (PRS) based on MDD‐GWAS data and demonstrated its performance in predicting depression onset in LOAD patients. Furthermore, using bulk brain transcriptomic data, we discovered sex‐dependent differentially expressed genes in AD comorbid with depression compared to AD only. Here we aimed to investigate in‐depth differences in gene expression on a cell‐type specific level between autopsied AD brain tissues from patients with depression compared to patients without evidence for depression.

**Method:**

Single nucleus (sn)RNA‐seq data from the ROSMAP study (available through AMP‐AD) was analyzed, specifically, dorsolateral pre‐frontal cortex datasets from 14 brains with AD comorbid depression and from 19 brains with AD and absence of depression. Depression was determined by clinical diagnosis and CES‐D scores. Differential gene expression (DEG) was performed using Nebula with case/control status based on clinical diagnosis of depression and age, sex, PMI and subject identified included as covariates.

**Result:**

FDR‐significant DEG were found for multiple cell types with the greatest majority of DEGs for inhibitory neurons (n = 548) followed by astrocytes (n = 109). Most of the genes in these cell types were upregulated. In addition, *MS4A6A* gene was found to be significantly upregulated in microglia, supporting our prior results showing the pleiotropic role of *MS4A6A* in both MDD and AD. Pathway analysis for the inhibitory neuron DEGs showed significant enrichment for terms related to mitochondrial function including: mitochondrial electron transport and mitochondrial ATP synthesis coupled with electron transport, suggesting mitochondrial dysfunction.

**Conclusion:**

The results demonstrated numerous depression‐associated cell‐type specific DEGs in AD brains. Our single‐cell analysis provides mechanistic insight into the risk to develop comorbid depression in AD and advances our understanding of shared molecular etiologies and dysregulated pathways between AD and MDD. This knowledge has a translational impact toward the identification of actionable targets as novel therapies to treat depression earlier in disease stages as a means to delay or alleviate depression onset in AD.

